# The Use of Metaraminol as a Vasopressor in Critically Unwell Patients: A Narrative Review and a Survey of UK Practice

**DOI:** 10.2478/jccm-2022-0017

**Published:** 2022-08-12

**Authors:** Lina Grauslyte, Nathalie Bolding, Mandeep Phull, Tomas Jovaisa

**Affiliations:** 1 Barts Health NHS Trust, London, UK; 2 Royal Free Hampstead NHS Trust, London, UK; 3 Barking Havering and Redbridge Hospitals NHS Trust, London, UK

**Keywords:** hypotension, metaraminol, shock, vasoconstrictor agents

## Abstract

**Background:**

Major international guidelines state that norepinephrine should be used as the first-line vasopressor to achieve adequate blood pressure in patients with hypotension or shock. However, recent observational studies report that in the United Kingdom and Australia, metaraminol is often used as second line medication for cardiovascular support.

**Aim of the study:**

The aim of this study was to carry out a systematic review of metaraminol use for management of shock in critically unwell patients and carry out a survey evaluating whether UK critical care units use metaraminol and under which circumstances.

**Methods:**

A systematic review literature search was conducted. A short telephone survey consisting of 6 questions regarding metaraminol use was conducted across 30 UK critical care units which included a mix of tertiary and district general intensive care units.

**Results:**

Twenty-six of thirty contacted centres responded to our survey. Metaraminol was used in 88% of them in various settings and circumstances (emergency department, theatres, medical emergencies on medical wards), with 67% reporting use of metaraminol infusions in the critical care setting. The systematic literature review revealed several case reports and only two studies conducted in the last 20 years investigating the effect of metaraminol as a stand-alone vasopressor. Both studies focused on different aspects of metaraminol use and the data was incomparable, hence we decided not to perform a meta-analysis.

**Conclusions:**

Metaraminol is widely used as a vasopressor inside and outside of the critical care setting in the UK despite limited evidence supporting its safety and efficacy for treating shock. Further service evaluation, observational studies and prospective randomised controlled trials are warranted to validate the role and safety profile of metaraminol in the treatment of the critically unwell patient.

## Introduction

Shock can be defined as an impairment of the normal relationship between oxygen demand and oxygen supply [1] and is the most frequent condition associated with critical care admission in the United Kingdom (UK). According to the Intensive Care National Audit and Research Centre (ICNARC) data, 89.9% of patients admitted to UK critical care units received basic cardiovascular support at some point during their stay and 16.8% of all patients received advanced support [2]. Advanced cardiovascular support implies that during their stay in critical care they: 1) received at least one vasoactive agent; 2) had continuous observation of cardiac output and derived indices; 3) had an intra-aortic balloon pump or other assist devices; 4) had a temporary pacemaker inserted [3]. Shock and associated conditions carry a mortality rate of up to 60% thus processes in the management of these patients are important to optimise for patient benefit [4].

Although vasoactive agents have been used and studied for more than six decades, the optimal choice of vasopressor remains uncertain [4, 5, 6]. Over the years, there have been significant changes in trends in vasoactive medication use [7]. High quality research led to a significant decline in the use of epinephrine, dopamine, and dobutamine as the first-line infusions and a shift towards norepinephrine and vasopressin as the first-and second-line vasoactive agents, respectively [8, 9, 10, 11, 12].

However, recent studies report that metaraminol is the second commonest first line vasopressor for the initial stages of cardiovascular support after noradrenaline [13]. This relatively recent trend is chiefly influenced by increasing early use of peripherally administered vasoactive agents. While numerous studies were published on the efficacy and safety of peripherally administered noradrenaline, it’s use in the United Kingdom is limited by prescribing and drug administration guidance stipulated in the British National Formulary (BNF). Administration through a central venous catheter is the only administration route listed in the BNF for noradrenaline [14], whereas no such restriction is listed for metaraminol [15]. As a result, peripheral administration of noradrenaline can be considered unlicensed or “off-label” use with potential medico-legal implications of such prescribing practices. The routes of administration listed in the Australian Injectable Drugs Handbook are identical to those in the BNF [16]. These national prescribing policies are likely to be a significant contributing factor to the widespread use of metaraminol in Australia and the United Kingdom.

Our aim was to conduct a systematic review and investigate the current evidence regarding the use of metaraminol for hypotension and shock in critical care and to conduct a survey to evaluate the circumstances of metaraminol use in critical care settings in the UK.

## Methods

### Systematic review

A literature search was conducted between the 3^rd^ and 4^th^ December 2021 using the terms ‘metaraminol’ OR ‘arginine’ in titles and abstracts of the articles. The following databases and search engines were used: Ovid Medline, Cochrane, PubMed and CINHAL. Articles we eligible for inclusion if they reported randomised trials or observational studies exploring the clinical effectiveness of metaraminol as a stand-alone vasopressor. Only articles reporting on adult patients and available in full text in English language were considered.

Articles were excluded if: (1) metaraminol was used for intraoperative hypotension in theatres; (2) trials were conducted more than 20 years ago; (3) metaraminol was used in conditions not related to hypotension or shock; (4) trials done in obstetric anaesthesia; (5) case reports; (6) metaraminol used alongside other vasoconstrictive medications; (7) trials focusing on dosing of metaraminol.

The time frame of 20 years for article exclusion was chosen to avoid the impact of major changes in all critical care practices (ventilation, sedation, invasive monitoring) that have taken place in the preceding decades [17,18].

The publication selection process was reported according to the Preferred Reporting Items for Systematic Reviews and Meta-Analyses (PRISMA) protocol [19]. All titles and abstracts were reviewed by two investigators independently (LG and TJ) and full texts were reviewed by one investigator (LG). During the search only two articles fitting the eligibility criteria were selected. The data they reported focused on different aspects of metaraminol use, hence a meta-analysis could not be performed. A narrative review was done instead.

### Survey

Our aim was to examine a broad sample of critical care units in the UK. According to the DEFINE database [20], 177 trusts in England currently use metaraminol. We investigated 30 departments, representing small (< 10 beds), medium (10-20 beds), and large (> 20 beds) units, and both teaching and non-teaching hospitals. We conducted a telephone survey of critical care doctors (specialty registrar and above) using a semi-structured narrative interview and a pre-defined list of questions (see Table 1).

**Table 1:** Survey questions used to collect data on metaraminol use in hospitals in United Kingdom

We are conducting a survey to evaluate the frequency and circumstances of metaraminol use in critical care settings in the UK. Your answers will not be discussed individually, and data will not be identifiable, as they will only be used as part of the whole sample.
1. Do you use metaraminol in the pre-critical care setting in your hospital?
2. If so, under which circumstances?
3. If metaraminol was started pre-ICU, is it continued in the critical care?
4. Do you ever use metaraminol as a first line vasopressor in Critical Care?
5. If so, under which circumstances?

## Results

### Narrative review

**Fig. 1:**
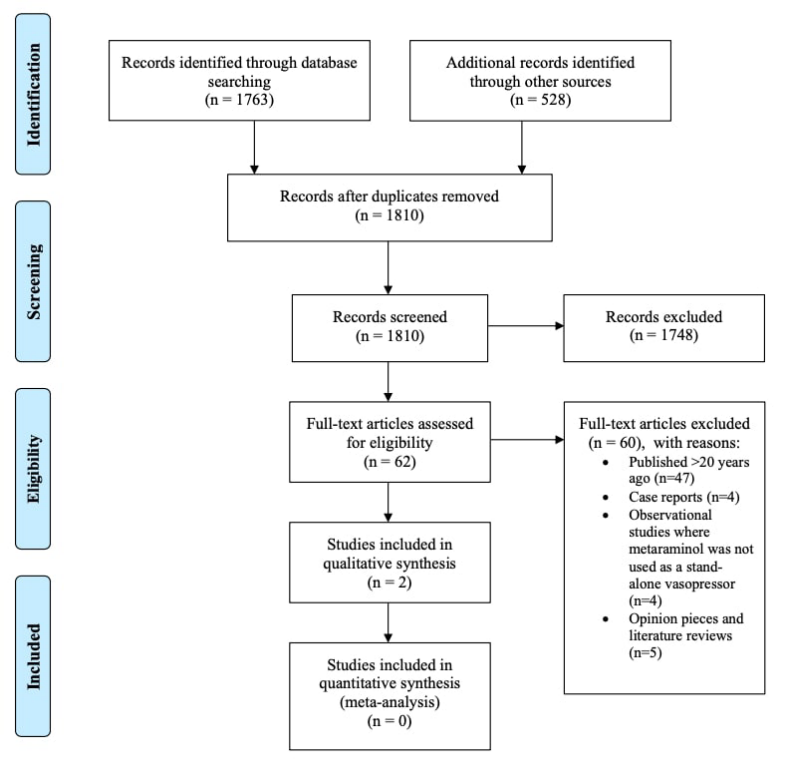
PRISMA flow chart of the study selection process

We identified 62 articles reporting the use of metaraminol for hypotension or shock in critical care settings (Figure 1).

47 articles (list in Appendix A) were excluded because they were published more than 20 years ago. Thirteen other articles that were excluded [21, 22, 23, 24, 25, 26, 27, 28, 29, 30, 31, 32], as they did not meet the inclusion criteria. Four were either prospective or retrospective observational studies regarding metaraminol use in combination with other vasopressors, metaraminol dosing, frequency of metaraminol use [21,23,24,33,34]. Four were case reports, highlighting use of metaraminol for shock of different etiologies [22,27,28,31]. Five articles were opinion pieces on current practice [26,29] and existing literature [25,30]. More detailed descriptions of the excluded articles are available in Appendix B.

There were no randomised controlled trials fitting the eligibility criteria identified during the literature search.

The two remaining studies [33,35] studies focused on different aspects of metaraminol use, rendering any statistical analysis impossible. Natalini et al. [35] carried out a prospective cohort analysis aiming to compare the haemodynamic effects of metaraminol and norepinephrine in patients with septic shock. Ten patients were enrolled in the study. All study participants had pulmonary artery catheters inserted to measure haemodynamic variables. The acid-base status and doses needed to achieve haemodynamic goals were also noted. No significant changes between effects of metaraminol and norepinephrine were observed.

Ruchti et al [33] published a letter on a prospective comparison of peripheral use of metaraminol and diluted norepinephrine. A hundred patient were included in the study without any randomisation. No statistically significant differences in demographics, vasopressor requirements, duration of treatment or side effects were observed. A basic economic analysis revealed that using metaraminol was significantly more costly than using peripheral norepinephrine.

Summary of both studies is provided in Table 2.

**Table 2:** Review of articles selected by our literature review regarding the use of metaraminol in critical care settings

Author and year of study	Type of the study	Number of patients and population	Outcome(s) evaluated	Narrative outcome	Quality assessment
Natalini et al, 2005(35)	Prospective cohort study	10 patients in with septic shock. All initially started on norepinephrine, then changed to metaraminol, dose titrated to reach same mean arterial blood pressure. Pulmonary catheter used for measurement of haemodynamic variables.	- Haemodynamic variables; - Medication doses needed to maintain same blood pressure; - Acid-base status - Cardiac output increase of more than 30%	No significant changes in haemodynamic variables. No relationship between norepinephrine and metaraminol doses (R^2^=0.087), needed to maintain the same mean arterial blood pressure was found.	The sample size was small. The study was not blinded. Only immediate effect was evaluated, and the authors did not comment on the effects that metaraminol can have on long term outcomes

Ruchti et al, 2021(33)	Prospective observational study	100 patients with shock or hypotension of any cause. First 50 given metaraminol, other 50- norepinephrine.	- Haemodynamic variables; - Duration of vasopressor support - Need of central venous catheter - Cost of treatment	No statistically significant changes in measured clinical outcomes were found in both groups. Cost of treatment was significantly higher in metaraminol group.	Patients were not randomised to intervention. Norepinephrine administered peripherally, which is not standard practice. Study presented as a letter, hence limited information available regarding methods and results.

### Survey results

We received 26 responses from the 30 hospitals we contacted, representing 10% of UK adult critical care units. Of these 4 were small (<10 bedded), 10 were medium (10-20 bedded), and 12 were larger sized (>20 bedded) units. The surveyed units had a collective total of 34206 admissions in 2018, representing 17% of total UK critical care admissions. The average number of admissions per small unit was 419/year, medium unit 757/year and larger unit 2080/year.

In total, 88% of the units (23/26 hospitals) used metaraminol in pre-critical care (prior to critical care admission). Of the three units that did not, two specified that metaraminol was only used when phenylephrine was not available, and the third one explained that it was never used because it was not available in their hospital.

In 70% (16/23) of the hospitals, metaraminol was used on the wards or in the emergency department for patients who were planned to be admitted to intensive care units. More details on the use of metaraminol in the pre-critical care setting provided in Table 3.

**Table 3:** Reported use of metaraminol in pre-critical areas as per the size of the critical care capacity

	All pre-critical care areas	Emergency department/resus	Theatres	Recovery	Ambulance/ Transfer	Wards
All respondent (26 hospitals)	23 (88%)	16 (70%)	9 (39%)	5 (22%)	1 (4%)	16 (70%)
<10 bedded (4 hospitals)	4 (100%)	4 (100%)	2 (50%)	1 (25%)	0	3 (75%)
10-20 bedded (10 hospitals)	9 (90%)	4 (40%)	2 (20%)	0	0	5 (50%)
>20 bedded (12 hospitals)	10 (83%)	8 (67%)	5 (42%)	4 (33%)	1 (8%)	8 (67%)

In 70% (16/23) of the cases, metaraminol was used to manage hypotension due to any cause. One hospital specified that it was used for sepsis-related hypotension, and another one used it for sedation-related hypotension only. Almost a quarter of all respondents explained that metaraminol was mostly used as a bridge to a central line and was discontinued as soon as norepinephrine could be started.

The majority of hospitals (67%, 15/23) continued metaraminol infusion in the critical care unit. Almost half of the respondents explained that infusion was time-limited (usually 12–24 hours). If cardiovascular support was required following this period of time, patients would be continued on an infusion of norepinephrine. In a few cases, continuation of metaraminol or the duration of its use depended on the doctor’s discretion.

**Fig. 2:**
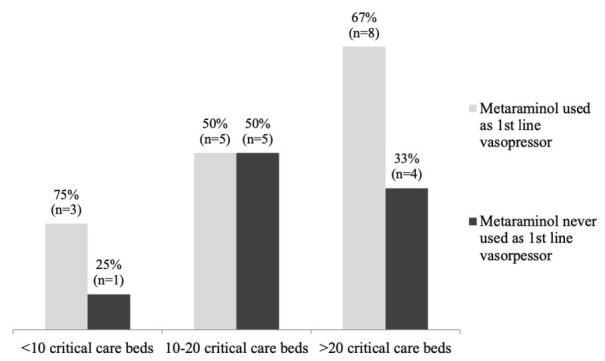
Use of metaraminol as first line vasopressor, in the critical care setting, based on the critical care capacity

Approximately 62% (14/23) of hospitals used metaraminol as first line vasopressor in critical care (Figure 2).

Two hospitals specified that metaraminol was used pre-intubation, four mentioned that it was used for sedation-related and post-operative hypotension on ICU. In one hospital it was the first line vasopressor for all causes of hypotension if patients were admitted or were treated in the high dependency unit. In two hospitals, metaraminol was used for hypotension due to any cause, if it was suspected to resolve relatively quickly on ICU.

## Discussion

Our survey has demonstrated that metaraminol is used commonly in pre-critical care settings and on the critical care units across the UK as the first-line vasopressor. Our literature review has demonstrated the lack of robust evidence to support this practice. The pharmacodynamic properties of metaraminol provide a rationale for its use, but there is no evidence to evaluate its impact on important patient outcomes, such as the length of vasopressor support, complications associated with its use, morbidity, and mortality. The pharmacological profile of metaraminol lends itself to its use as a vasopressor which can be quickly drawn up and administered via a peripheral cannula to stabilise an acutely hypotensive shocked patient. It is a synthetic, direct and indirect sympathomimetic agonist mainly for alpha-1 adrenoreceptors, but also for some betaadrenoreceptors [36]. The frequency of adverse events with metaraminol has not been clearly established. Both observational studies that we included in the narrative review [33,35] demonstrated, that metaraminol is able to sustain arterial pressure while producing global haemodynamic parameters near identical to those of noradrenaline. The most informative study to date by Natalini et al used the cross-over design in a small group of patients allowing a direct comparison of metaraminol and noradrenaline effects in the same individual. Considering that cardiac index and stroke volume index were identical with identical markers of preload and afterload (central venous pressure, pulmonary artery occlusion pressure and systemic vascular resistance index), it would be feasible to conclude that both metaraminol and noradrenaline have very similar pattern of alpha and beta adrenergic activity. Of note, a marginally higher mean pulmonary artery pressure and pulmonary vascular resistance index was recorded following initiation of metaraminol therapy. Such an effect may be of clinical importance in patients with pre-existing pulmonary hypertension or cases where pulmonary artery pressure is elevated due to primary pathological processes in the lung or lung vasculature. Strikingly, there was no correlation in the dose required to achieve same haemodynamic parameters, suggesting that there is significant contribution from the indirect action of metaraminol on adrenergic receptors. It is therefore possible that metaraminol may be a useful agent in patients with shock refractory to norepinephrine. However, this paper did not report the duration of metaraminol therapy, thus it is impossible to state at present if the same haemodynamic effects would be sustained over a prolonged infusion time measured in days rather than hours. Neither of the studies were randomised or had a clinically relevant endpoint so they cannot inform patient care. Without randomised controlled trials it is impossible to balance the risk and benefit for the patients.

Our survey demonstrates that while indications for initiation of metaraminol as a first-line vasopressor may be rational, reasons for discontinuation are very arbitrary and are often based on clinical decisions of individual clinicians.

It is plausible, though not the only reason, that the usability of metaraminol for critically ill patients may have stemmed from its use in anaesthesia; an ideal, short acting peripherally administered vasopressor for episodic or short periods of hypotension related to anaesthetic drugs. Whilst this may be so, the critically unwell patient is physiologically different from the surgical patient presenting for elective surgery [37,38].

In addition to patient outcomes, we should consider the financial impact of the increasing use of metaraminol in critical care. One of the large units in this survey reported that the total cost of metaraminol exceeded that of norepinephrine in the 2018-2019 financial year (£43,717 v £29,662), making it the 4^th^ most expensive drug used in that unit. Costs are likely to be significant once extrapolated to the wider NHS level. It is possible that some of that cost of using metaraminol could potentially be offset by the reduced use of central venous catheters (CVC). However, often CVCs are inserted for multiple reasons beside the use of vasopressors or inotropes; thus, the use of peripheral vasopressor may not necessarily produce significant reduction in the use of CVCs.

The extent of metaraminol use, lack of evidence and potential financial impact on critical care budgets warrant further research. We only need to look to a similar agent phenylephrine where, despite biological plausibility, studies indicated potential harmful outcomes when this was used in critically unwell patients [39]. Additionally, in recent clinical trials, some of the newer vasopressors such as selepressin failed to produce expected clinical benefit [6].

Randomised controlled trials are needed to validate the use and role of metaraminol in critically unwell patients. Our survey demonstrates significant heterogeneity of indications and uses of metaraminol such that multiple questions arise, for example, what is the role of peripheral vasopressors in ICU and how do they fare against centrally administered agents for patient related outcomes. Metaraminol has been used widely in our centre for well over a decade, with increasing incidence of it’s use as a first line vasopressor. While this is in line with national trends demonstrated in our survey, it is worth noting that variability in practice is notably not only amongst institutions, but also amongst intensive care physicians working on the same unit. In our opinion this would indicate a sufficient degree of equipoise amongst medical professionals to make a randomised controlled trial ethically feasible. Our survey has identified clinical trends but was not designed to highlight specific patterns or look at outcomes. It is unclear which indications would attract sufficient equipoise to enable a well-designed, clinically relevant and applicable randomised trial. We suggest that further research should start with a prospective observational multicentre study, which would allow to gain quantitative information on vasopressor use. This would allow researchers to formulate hypotheses and trial questions for randomised studies in the future.

## Conclusions

Metaraminol was the 1^st^ line vasopressor in a representative sample of UK critical care units. We identified no randomised trials describing clinical outcomes of metaraminol use, meaning the benefits and risks of this treatment are uncertain. Further observational studies and prospective randomised controlled trials are warranted to inform evidence-based practice for patient benefit.

## Appendices

### Appendix A

Articles that were excluded, because they published 40-60 years ago:

- Moyer JH, Mills LC. Vasopressor agents in shock. Am J Nurs 1975; 75: 620-5).
- Marsden DE, Cavanagh D. Hemorrhagic shock in the gynecologic patient. Clin Obstet Gynecol 1985; 28: 381-90.
- Smith HJ, Oriol A, Morch J, McGregor M. Hemodynamic studies in cardiogenic shock. Treatment with isoproterenol and metaraminol. Circulation 1967; 35: 1084-91.
- Tristani FE, Cohn JN. Studies in clinical shock and hypotension. VII. Renal hemodynamics before and during treatment. Circulation 1970; 42: 839-51.
- Sambhi MP, Weil MH, Udhoji VN, Rosoff L. Effect of pressor amines on cardiac output in patients with acute hypotension. Circulation 1965; 30: 485-92.
- Cavanagh D, Rao PS, Roberts WS. Septic shock in the gynecologic patient. Clin Obstet Gynecol 1985; 28: 355-64.
- Weil MH. Clinical studies on a vasopressor agent: metaraminol (Aramine): III. Observations on its oral use in the treatment of hypotension. Am J Med Sci 1957; 233: 367-74.
- Keeley BR. Septic shock. Crit Care Quarterly 1985; 7: 59-67.
- Smith NT, Corbascio AN. The use and misuse of pressor agents. Anesthesiology 1970; 33; 58-101.
- Martinez JT, Fernandez G, Vazquez-Leon H. Clinical evaluation of new therapeutic concepts in septic shock. Obstet Gynecol 1966; 27: 296-301.
- Duff JH, Scott HM, Peretz DI, Mulligan GW, Maclean LD. The diagnosis and treatment of shock in man based on hemodynamic and metabolic measurements. J Trauma 1966; 6: 145-156.
- Sambhi MP, Weil MH, Udhoji VN, Rosoff L. Effect of pressor amines on cardiac output in patients with acute hypotension. Circulation 1964; 30: 485-92.
- Brown RS, Carey JS, Mohr PA, Monson DO, Shoemaker WC. Comparative Evaluation of Sympathomimetic Amines in Clinical Shock. Circulation 1966; 34: 260-71.
- Spoerel WE, Seleny FL, Williamson RD. Shock caused by continuous infusion of metaraminol (Aramine). Surv Anesthesiol 1965; 9: 32-33.
- Mills LC, Voudoukis I, Moyer JH, Heider C. Treatment of shock with sympathicomimetic drugs: use of comparison with other vasopressor agents. Surv Anesthesiol 1962; 6: 18.
- Weil MH. Current Concepts on the Management of Shock. Circulation. 1957; 16: 1097-105.
- Weil MH. Clinical studies on a vasopressor agent: metaraminol (Aramine). II Observations on its use in the management of shock. Am J Med Sci 1955; 230: 357-69.
- Udhoni VN, Weil MH, Sambhi MP. Pressor amines and angiotensin in the treatment of shock. Int Anesthesiol Clin 1964; 2: 399-419.
- Stechel GH, Fishman SI, Schwartz G, Turkovitz H, Madonia PF, Fankhauser A. The Use of Aramine in Clinical Shock. Circulation 1956; 13: 834-6.
- Sagie A, Sclarovsky S, Klainman E, Strasberg B, Rechavia E, Mager A et al. Effect of metaraminol during acute inferior wall myocardial infarction accompanied by hypotension: preliminary study. J Am Coll Cardiol 1987; 10: 1139-44.
- Cohn JN, Luria MH. Studies in clinical shock and hypotension. 3. Comparative effects of vasopressor drugs and dextran. Arch Intern Med 1965; 116: 562-6.
- Mills LC, Steppacher R. Hemodynamic effects of vasopressor agents in hemorrhagic shock. Am J Cardiol 1963: 12: 614-8
- Binder MJ. Effect of vasopressor drugs on circulatory dynamics in shock following myocardial infarction. Am J Cardiol 1965; 16: 834-40.
- Kuhn LA. The treatment of cardiogenic shock. II. The use of pressor agents in the treatment of cardiogenic shock. Am Heart J 1967; 74: 725-8.
- Weil MH, Shubin H, Biddle M. Shock caused by gram-negative microorganisms. Analysis of 169 cases. Ann Intern Med 1964; 60: 384-400.
- Bourdarias JP, Dubourg O, Gueret P, Ferrier A, Bardet J. Inotropic agents in the treatment of cardiogenic shock. Pharmacol Ther 1983; 22: 53-79.
- Moyer JH, Beazley HL. Effectiveness of aramine in the treatment of shock. Am Heart J 1955; 50: 136-44.
- Shubin H, Weil MH. Practical considerations in the management of shock complicating acute myocardial infarction. A summary of current practice. Am J Cardiol 1970; 26: 603-8.
- Figueras J, Lidon RM, Cortadellas J. Metaraminol-induced reversal of acute myocardial ischaemia associated with hypotension and refractory to intravenous nitroglycerin. Eur Heart J 1991; 12: 720-5.
- Smith HJ, Oriol A, Morch J, McGregor M. Hemodynamic studies in cardiogenic shock. Treatment with isoproterenol and metaraminol. Circulation 1967; 35: 1084-91.
- Takkunen J, Oilinki O, Huhti E, Vuopala U, Koivisto O. Medical tretment and mortality in cardiogenic shock. Metaraminol compared with combined phentolamine and norepinephrine. Glucagon therapy. Acta Med Scand 1972; 192: 165-70.
- Agress CM. Therapy of cardiogenic shock. Prog Cardiovasc Dis 1963; 6: 236-54.
- Kones RJ. Cardiogenic shock: therapeutic implications of altered myocardial energy balance. Angiology 1974; 25: 317-33.
- Hardaway RM, James PM Jr, Anderson RW, Bredenberg CE, West RL. Intensive study and treatment of shock in man. JAMA 1967; 199: 779-90.
- • Hermreck AS, Thal AP. The adrenergic drugs and their use in shock therapy. Curr Probl Surg 1968; 1-46.
- Mir MA, Rees S, Yahya AM, Williams JD, Reeves TL. Clinical appraisal of shock following acute myocardial infarction. Cardiology. 1974; 59: 69-82.
- Walters MB. Drugs for shock following acute myocardial infarction. Can Med Assoc J. 1965; 93: 933-4.
- Aviado DM. Pharmacologic approach to the treatment of shock. Ann Intern Med 1965; 62: 1050-9.
- Smulyan H, Cuddy RP, Eich RH. Hemodynamic effects of pressor agents in septic and myocardial infarction shock. JAMA. 1964; 190: 188-94.
- Ruedy J, Dirks JH, Cameron DG. Bacteremic shock— a medical emergency. Can Med Assoc J 1963; 89: 1059-63.
- Besterman EM. Treatment of cardiac shock by metaraminol. Br Med J 1959; 1: 1081-3.
- Udhoji VN, Weil MH. Circulatory effects of angiotensin, levarterenol and metaraminol in the treatment of shock. N Engl J Med 1964; 270: 501-5.
- Cavanagh D, McLeod AG. Septic shock in obstetrics and gynecology. An evaluation of metaraminol therapy. Am J Obstet Gynecol 1966; 96: 913-8.
- Weil MH, Shubin H. Treatment of shock caused by bacterial infections. Calif Med 1973; 119: 7-13.
- Moyer JH, Handley CA. Blood pressure and renal hemodynamic responses to aramine and the alterations of these responses by adrenergic blockade with dibenzyline. Am Heart J 1954; 48: 173-84.
- Moyer JH, Mills LC. Vasopressors for treating shock. Postgrad Med 1974; 56: 172-80.
- Corday E, Lillehei RC. Pressor agents in cardiogenic shock. Am J Cardiol 1969; 23: 900-10.

### Appendix B

Articles that were excluded after screening.

**Table:**

Author and year published	Type of article	Main medical condition reported	Comments on metaraminol use
Hou et al, 2007 (24)	Retrospective observational cohort study	Patients admitted to the intensive care units with septic shock	Metaraminol used together with dopamine. Haemodynamic variables and impact on renal function evaluated as outcome measures.

Abu Sardaneh et al, 2021 (34)	A retrospective cohort study to determine the dose equivalent between metaraminol and noradrenaline	Patients with shock of any cause in the intensive care unit.	The conversion dose ratio between continuous infusion metaraminol and norepinephrine ranged between 8 and 13 in the primary and sensitivity analyses.

Abu Sardaneh et al., 2021 (21)	A retrospective observational study	Patients with shock of any cause in the intensive care unit.	Metaraminol was the most commonly used first line vasopressor. The study reported on the practice and did not focus on outcomes.

Abu Sardaneh et al, 2021 (23)	A retrospective matched observational study	Patients with shock of any cause in the intensive care unit.	In critically ill patients, metaraminol used alongside norepineohrine may be associated with a longer time to resolution of shock compared with those who only receive norepinephrine.

Da Silva and Furtado, 2018 (22)	A case report	Anaphylactic shock	Metaraminol mentioned as a vasopressor in shock

Anderson and Chatha, 2017 (25)	Literature review	Metaraminol use in the Emergency Department	Concluded that it is widely used in the emergency department setting with little evidence to support the practice.

Hayward et al, 2016 (26)	A review of practice	Review of medications used by Australian aeromedical prehospital and retrieval service.	Metaraminol only briefly mentioned as a treatment option utilised by the Australian pre-hospital service

Redmond et al, 2013 (27)	A case report	Takotsubo cardiomyopathy	A patient, suffering from at that point undiagnosed Takotsubo cardiomyopathy progressed to pulseless ventricular tachycardia following bolus administration of metaraminol.

Isbister and Duffull, 2009 (28)	A case report	Quetiapine overdose	Metaraminol used successfully to maintain blood pressure (as opposed to conventional vasopressors)

Dewachter et al, 2007 (29)	A review of current practice	Perioperative anaphylaxis	Metaraminol briefly mentioned as a vasopressor used to treat hypotension

Brown, 2005 (30)	Review of management of anaphylaxis	Anaphylactic shock	Metaraminol briefly mentioned as a vasoconstrictor in shock.

Wood et al, 2005 (31)	A case report	Amlodipine overdose	Metaraminol used successfully to maintain blood pressure (as opposed to conventional vasopressors)

Holmes, 2005 (32)	Opinion piece	Opinion piece on vasopressors in the Intensive Care	Metaraminol mentioned alongside other vasopressors used to treat hypotension
